# Supplementary material for: Functional antagonism between ΔNp63α and GCM1 regulates human trophoblast stemness and differentiation
Source: Nat Commun. 2022 Mar 25;13:1626. doi: 10.1038/s41467-022-29312-6 (PMC8956607; doi:10.1038/s41467-022-29312-6)
Supplement: Supplementary file 1 — Supplementary Information [file 41467_2022_29312_MOESM1_ESM.pdf]

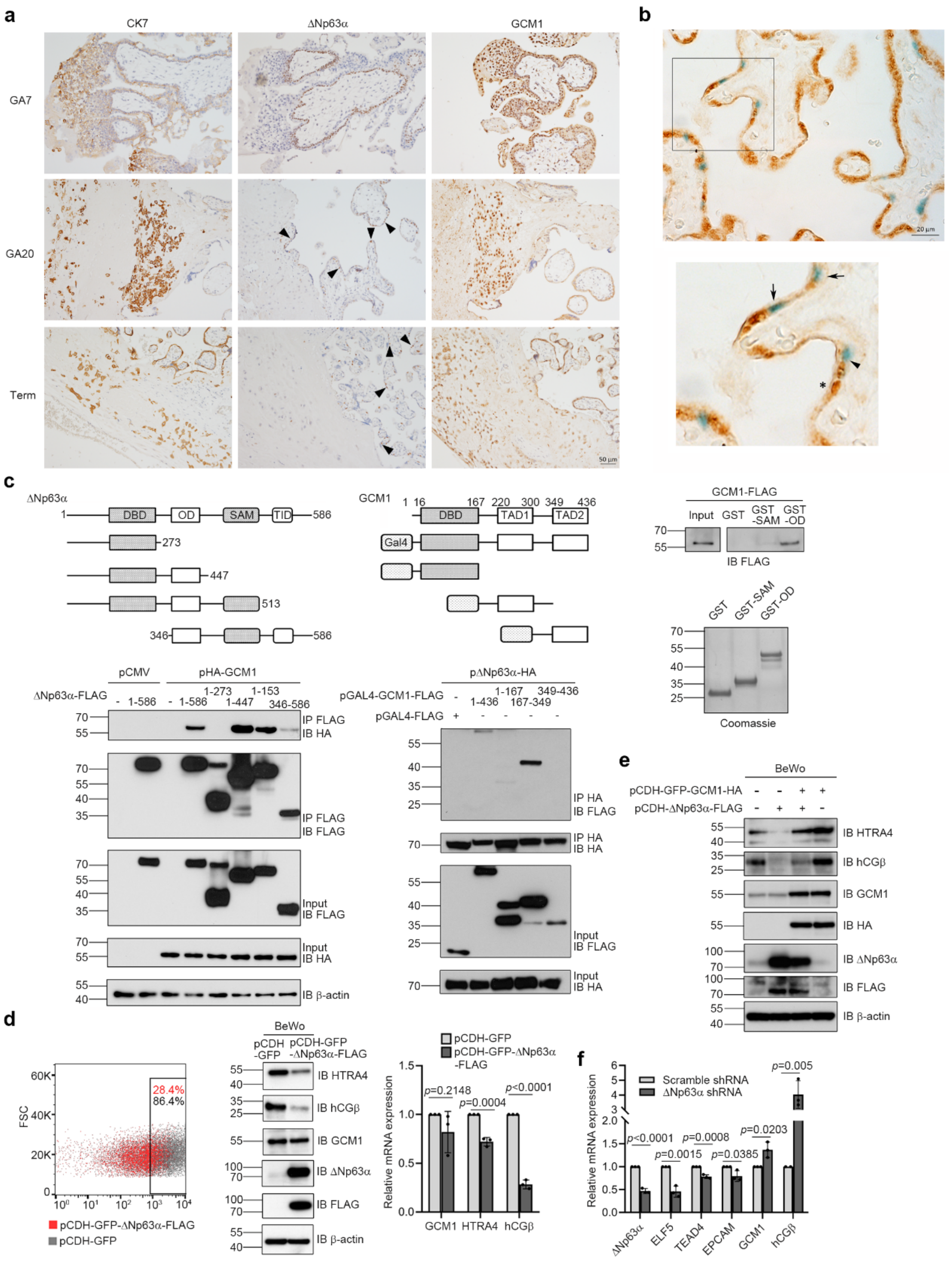

Supplementary Figure 1. Expression of ΔNp63α and GCM1 in the human placenta and characterization of the ΔNp63α-GCM1 interaction. **a,** Immunohistochemistry of ΔNp63α and GCM1 in placentas at different gestational ages. Sections of first-trimester (gestational age 7 weeks, GA7), second-trimester (GA20), and term human placentas were immunostained with cytokeratin 7 (CK7), ΔNp63α, and GCM1 Abs, respectively. The sections were further counterstained with hematoxylin to localize the cell nuclei. Arrowhead indicates a ΔNp63α-expressing CTB or nucleus in the STB layer. Scale bar:

50 μm. **b,** Co-expression of ΔNp63α and GCM1 in placental trophoblasts. Term human placental sections were subjected to immunostaining using ΔNp63α Ab, AP-conjugated secondary Ab, and StayGreen chromogen (Abcam, Cambridge, UK), and then GCM1 Ab, HRP-conjugated secondary Ab, and DAB chromogen (Vector Labs, Burlingame, CA). A higher magnification image of the boxed region is shown below. Arrowhead, asterisk, and arrow indicate trophoblasts expressing ΔNp63α (green), GCM1 (brown), and both factors, respectively. Scale bar, 20 μm. **c,** Mapping of GCM1-interacting domain in ΔNp63α (left) and ΔNp63α-interacting domain in GCM1 (middle). 293T cells were transfected with the indicated expression plasmids, followed by coimmunoprecipitation analyses with FLAG and HA mAbs. DBD, DNA-binding domain; OD, oligomerization domain; SAM, sterile alpha motif; TID, transactivation inhibitory domain; TAD, transactivation domain. Direct interaction between GCM1 and ΔNp63α in pull-down assays (right). Recombinant GCM1-FLAG purified from 293T cells transfected with pGCM1-FLAG was incubated with bacterially expressed GST-ΔNp63α-SAM or GST-ΔNp63α-OD pre-bound to glutathione-conjugated agarose beads (GE Healthcare Biosciences, Pittsburgh, PA), followed by immunoblotting with FLAG mAb. **d,** Suppression of GCM1 target gene expression by ΔNp63α. BeWo cells were transduced with lentiviruses harboring pCDH-GFP or pCDH-GFP-ΔNp63α-FLAG, followed by flow cytometry of GFP-positive cells. Mock or ΔNp63α-FLAG-expressing BeWo cells were subjected to immunoblotting and quantitative RT-PCR analyses of GCM1, hCGβ, and HTRA4 proteins and transcripts. **e,** GCM1 overexpression counteracts the suppression of GCM1 target genes by ΔNp63α. BeWo cells were transduced with lentiviruses harboring pCDH or pCDH-ΔNp63α-FLAG, followed by puromycin selection. Mock or ΔNp63α-FLAG-expressing BeWo cells were further transduced with lentiviruses harboring pCDH-GFP or pCDH-GFP-GCM1-HA and subjected to immunoblotting analysis of GCM1, hCGβ, and HTRA4 proteins. **f,** Downregulation of TS stemness genes by *ΔNp63α* knockdown. Scramble control or *ΔNp63α*-knockdown JEG3 cells were subjected to quantitative RT-PCR analysis of the TS cell marker genes, *ELF5*, *TEAD4*, and *EPCAM*. Note that expression of *GCM1* and *hCGβ* genes are upregulated in the *ΔNp63α*-knockdown cells. Data are presented as mean ± SD of independent experiments (n=3 in **d**, **f**). Differences were assessed by unpaired two-tailed Student's *t*-test. Source data are provided in the Source Data file.

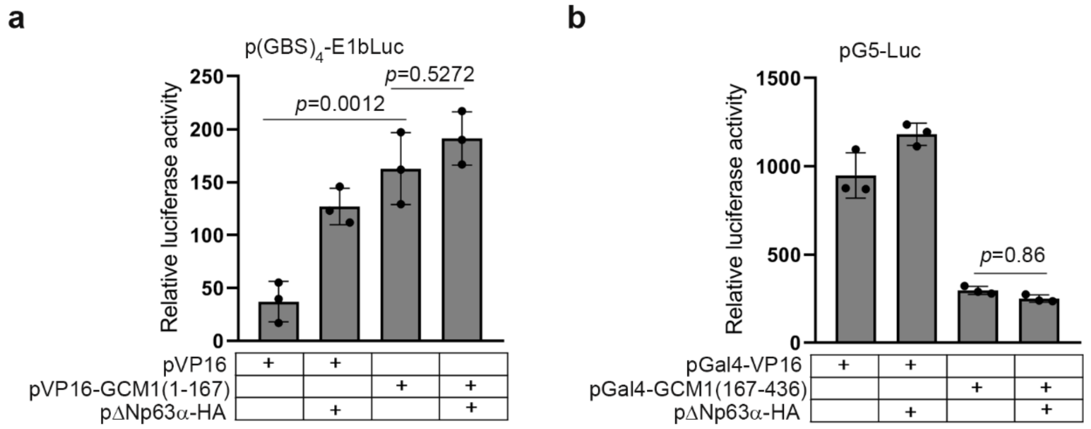

Supplementary Figure 2. ΔNp63α does not directly regulate the DNA-binding and transcriptional activities of GCM1. **a,** 293T cells were transfected with the GCM1 reporter plasmid p(GBS)$_4$-E1bLuc, the pVP16 plasmid encoding the VP16 TAD, the pVP16-GCM1(1-167) plasmid encoding a fusion protein of GCM1 DBD and VP16 TAD, and pΔNp63α-HA for assessing the effect of ΔNp63α on the DNA-binding activity of GCM1. **b,** 293T cells were transfected with the Gal4 reporter plasmid pG5-Luc, the pGal4-VP16 plasmid encoding a fusion protein of Gal4 DBD and VP16 TAD, the pGal4-GCM1(167-436) encoding a fusion protein of Gal4 DBD and GCM1 TAD, and pΔNp63α-HA for assessing the effect of ΔNp63α on the transcriptional activity of GCM1. Cells were harvested for luciferase assays at 48 h post-transfection. Data are presented as mean ± SD of independent experiments (n=3 in **a**, **b**). Differences were assessed by two-way ANOVA with Tukey's post hoc test. Source data are provided in the Source Data file.

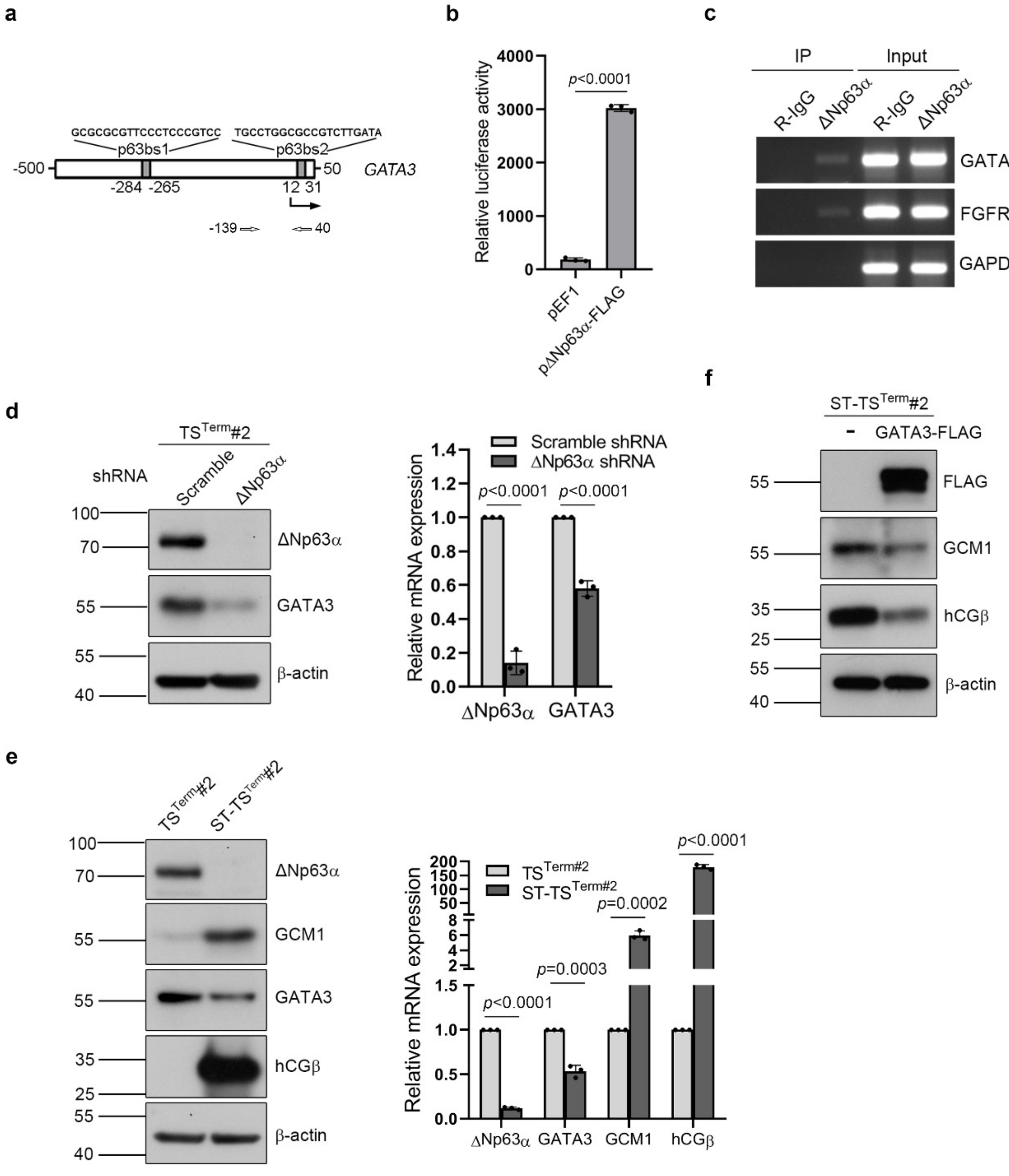

Supplementary Figure 3. Regulation of GATA3 promoter activity by ΔNp63α and regulation of STB differentiation by GATA3. **a,** Schematic representation of GATA3 promoter. Two candidate p63-binding sites (p63bs1, and -2) are listed. Arrow indicates the transcriptional start site. Arrows with transparent background indicate the ChIP primer set. **b,** ΔNp63α stimulates GATA3 promoter activity. The luciferase reporter plasmid pGATA3-0.5Kb, which harbors the 0.5 Kb genomic fragment upstream of the transcriptional start site of *GATA3*, was cotransfected with pEF1 or pΔNp63α-FLAG into Hep3B cells. At 48 h post-transfection, cells were harvested for luciferase assays. **c,** Association of ΔNp63α with *GATA3* promoter. TS^{Term}#2 cells were subjected to ChIP analysis using normal rabbit IgG (R-IgG) or a rabbit anti-p63α mAb (Cell Signaling). The immunopurified genomic fragments were analyzed by PCR with primer sets

specific for *GATA3* and the known p63 target gene, *FGFR2* [1]. The sequences of the primer sets were listed in Supplementary Table 1. **d,** Regulation of GATA3 expression by ΔNp63α in TS^{Term} cells. TS^{Term}#2 cells were transduced with lentivirus harboring scramble or ΔNp63α shRNA and cultured for 96 h and then subjected to immunoblotting and quantitative RT-PCR analyses of GATA3 and ΔNp63α proteins and transcripts. **e,** Downregulation of ΔNp63α and GATA3 during STB differentiation. TS^{Term}#2 cells were treated with FSK for 72 h for ST-TS^{Term}#2 differentiation and then subjected to immunoblotting and quantitative RT-PCR analyses of ΔNp63α, GCM1, GATA3, and hCGβ proteins and transcripts. **f,** Suppression of STB differentiation by GATA3. TS^{Term}#2 cells were transduced with lentivirus harboring GATA3-FLAG and treated with FSK for 48 h for ST-TS^{Term}#2 differentiation. Cells were then harvested for immunoblotting analysis of GATA3-FLAG, GCM1, and hCGβ proteins. Data are presented as mean ± SD of independent experiments (n=3 in **b**, **d**, **e**). Differences were assessed by unpaired two-tailed Student's *t*-test. Source data are provided in the Source Data file.

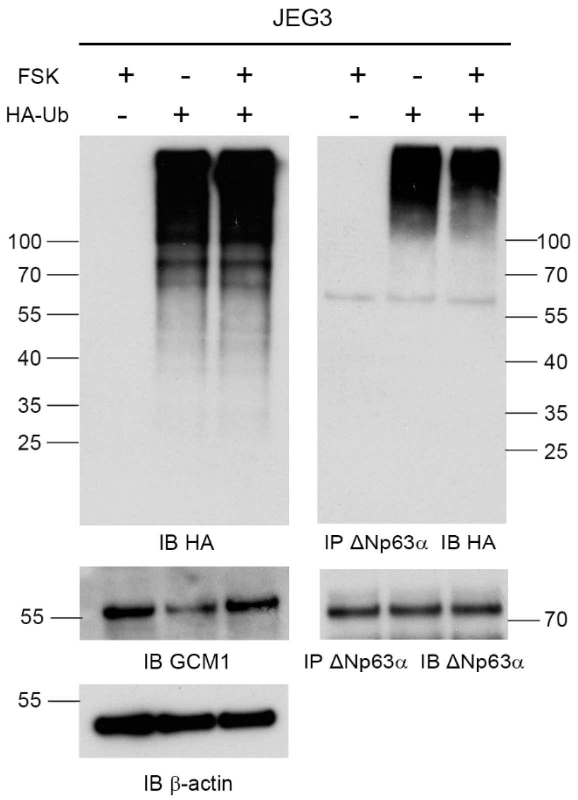

Supplementary Figure 4. Neither GCM1 nor FSK affects ΔNp63α ubiquitination. JEG3 cells were transfected with pHA-Ub, which is an expression plasmid encoding HA-tagged ubiquitin. At 24 h post-transfection, cells were treated with 5 μM MG132 plus or minus 50 μM FSK for another 12 h. Cells were then harvested for coimmunoprecipitation analyses with a guinea pig anti-p63α Ab and HA mAb. Note that levels of HA-Ub-conjugated ΔNp63α are not significantly affected by FSK or GCM1. Source data are provided as a Source Data file.

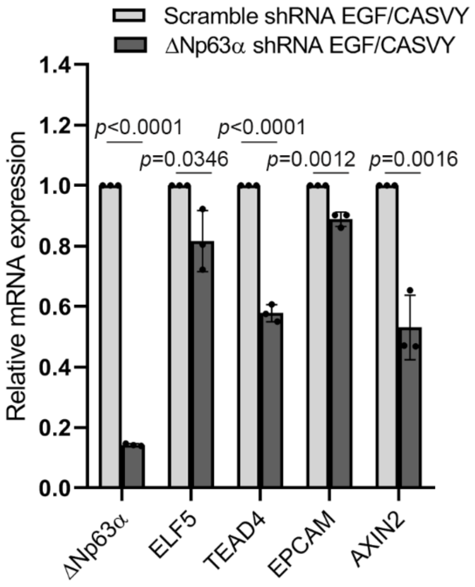

Supplementary Figure 5. Effects of *ΔNp63α* knockdown on EGF/CASVY-induced trophoblast stemness gene expression. Scramble control or *ΔNp63α*-knockdown BeWo cells were treated with EGF/CASVY for 24 h for analysis of trophoblast stemness gene expression. Because ΔNp63α enhances WNT signaling [2], *ΔNp63α* knockdown led to decreased *AXIN2* expression. Data are presented as mean ± SD of independent experiments (n=3). Differences were assessed by unpaired two-tailed Student's *t*-test. Source data are provided in the Source Data file.

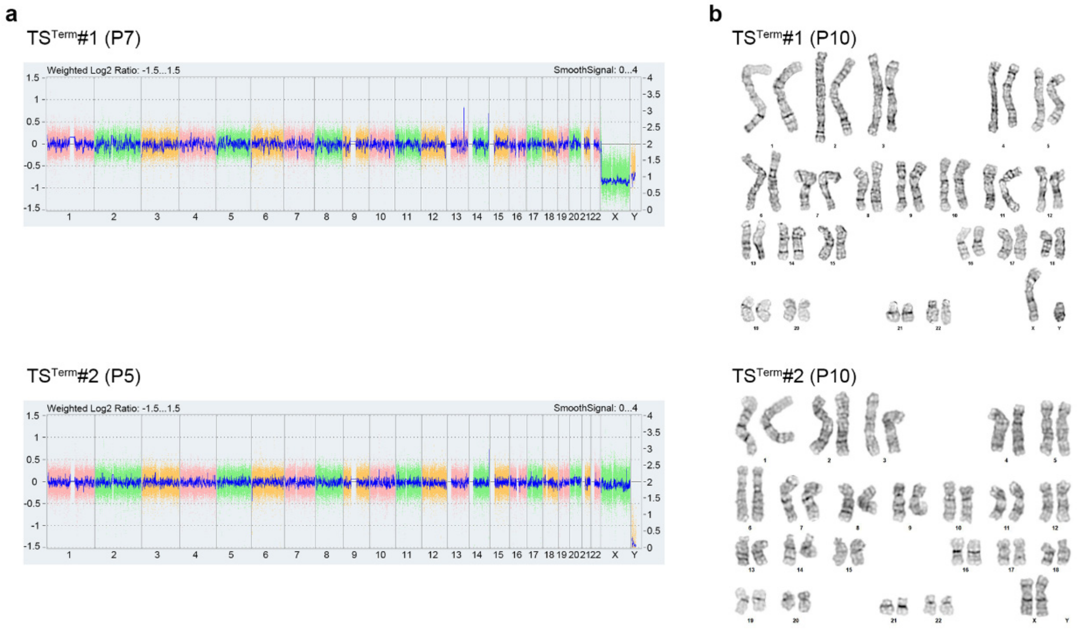

Supplementary Figure 6. Chromosomal analysis of TS^{Term} cells. **a,** Chromosomal microarray analysis of TS^{Term}#1 (P7) and #2 (P5) cells using the Affymetrix CytoScan 750K arrays. Data are expressed as the weighted log2 ratio of the copy number on the left Y-axis and the chromosome number on the X-axis. **b,** G-banded karyotyping of TS^{Term}#1 (P10) and #2 (P10) cells in metaphase.

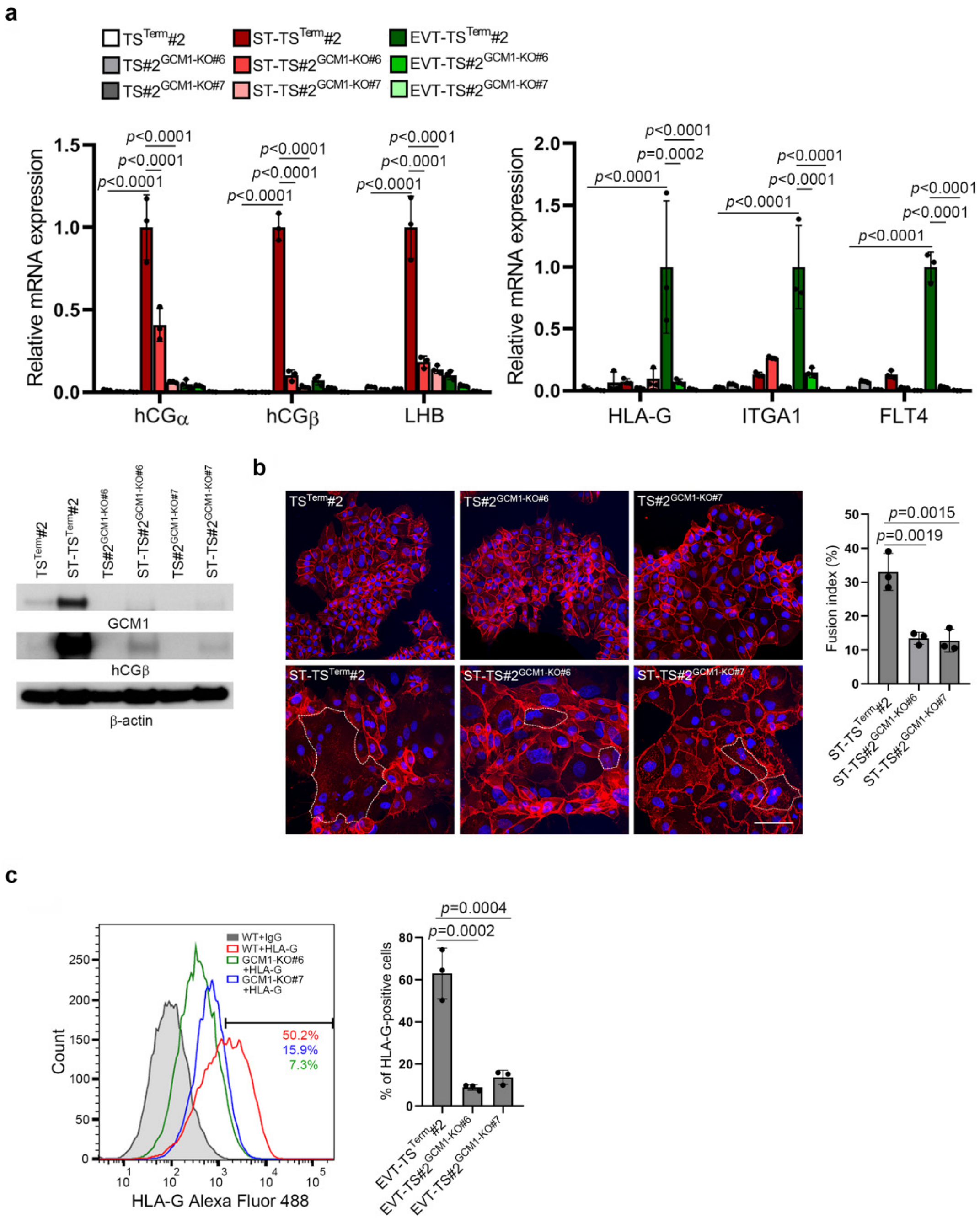

Supplementary Figure 7. GCM1 is essential for STB and EVT differentiation from TS^{Term} cells. **a,** WT and *GCM1*-KO TS^{Term} (TS#2$^{GCM1-KO#6}$ and TS#2$^{GCM1-KO#7}$) cells were induced into STBs or EVTs and then harvested for analysis of differentiation genes. Cell fusion (**b**) and surface expression of HLA-G (**c**) are impaired in the *GCM1*-KO TS^{Term} cells. Syncytial margins are marked with stippled line. Scale bar, 100 µm. Data are presented as mean ± SD of independent experiments (n=3 in **a-c**). Differences were assessed by two-way (**a**) or one-way (**b**, **c**) ANOVA with Tukey's post hoc test. Source data are provided in the Source Data file.

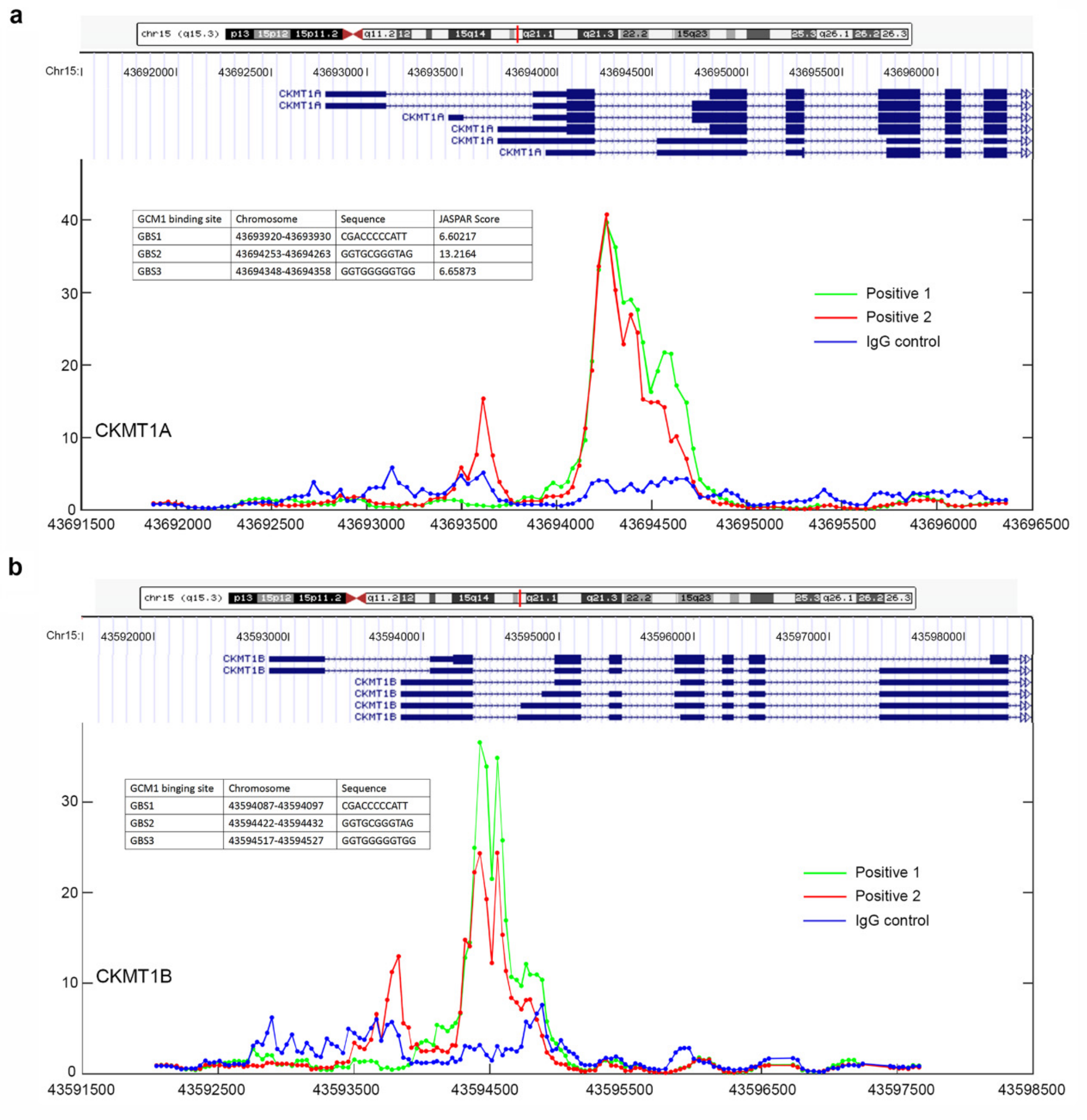

GCM1 binding site	Chromosome	Sequence	JASPAR Score
GBS1	43693920-43693930	CGACCCCCATT	6.60217
GBS2	43694253-43694263	GGTGCGGGTAG	13.2164
GBS3	43694348-43694358	GGTGGGGGTGG	6.65873

GCM1 binging site	Chromosome	Sequence
GBS1	43594087-43594097	CGACCCCCATT
GBS2	43594422-43594432	GGTGCGGGTAG
GBS3	43594517-43594527	GGTGGGGGTGG

Supplementary Figure 8. *CKMT1* is a GCM1 target gene. ChIP-chip experiments in BeWo cells stably expressing HA-GCM1 using HA mAb (positive) or normal mouse IgG (control) revealed association of HA-GCM1 with an intron region immediately downstream of exon 1 in the *CKMT1A* (**a**) and *-1B* (**b**) genes on chromosome 15q15.3. Putative GCM1-binding sites (GBSs) and their sequences are listed.

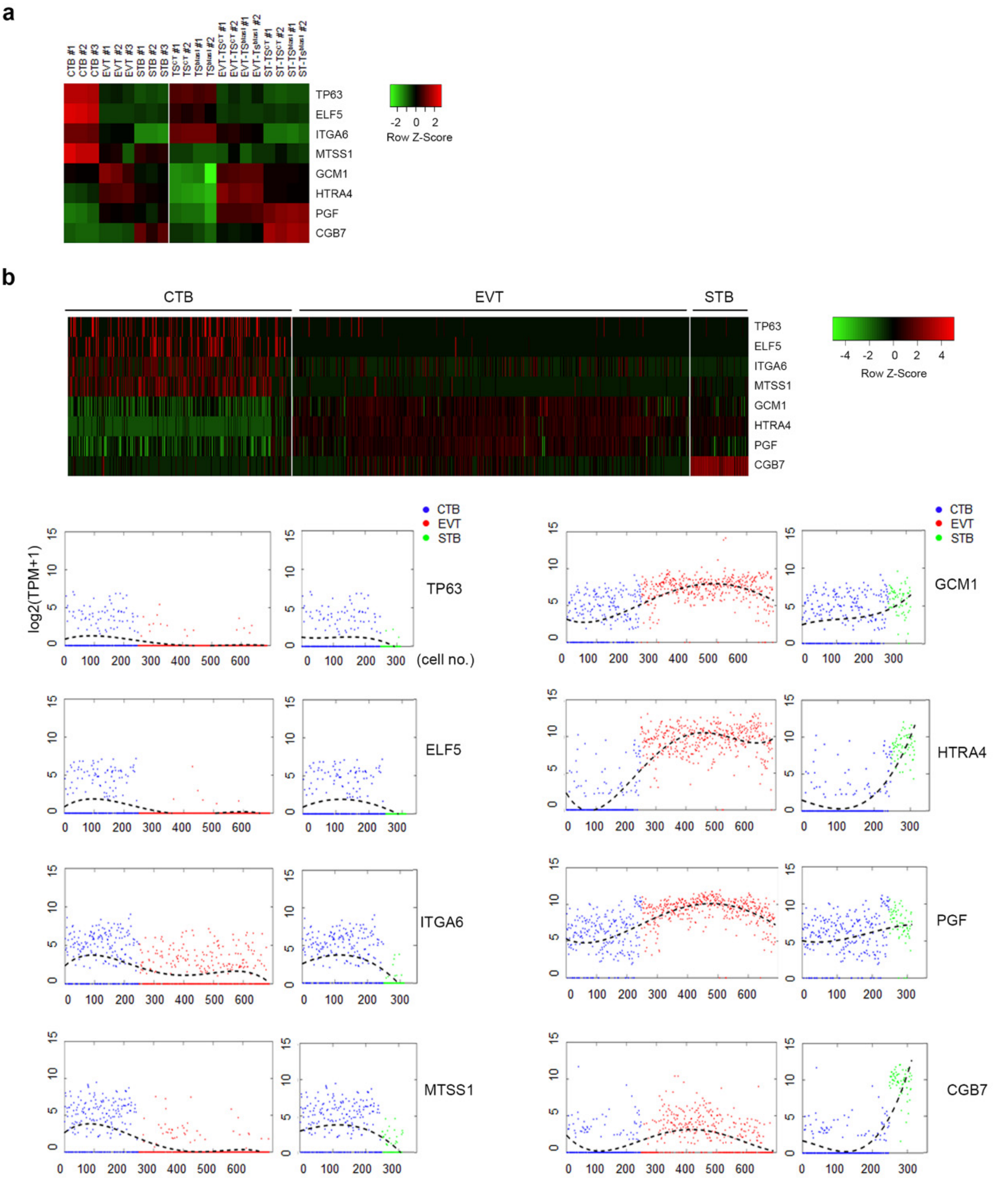

Supplementary Figure 9. Reciprocal expression of *GCM1* and *ΔNp63α* genes in $\mathrm{TS}^{\mathrm{CT}}$ and $\mathrm{TS}^{\mathrm{blast}}$ cells and differentiated trophoblasts. **a,** Meta-analysis of trophoblast differentiation and stemness gene expression in RNA-seq datasets (JGAD000073 and JGAD000115) of $\mathrm{TS}^{\mathrm{CT}}$ and $\mathrm{TS}^{\mathrm{blast}}$ cells and their derivative STBs (ST-TS cells) and EVTs (EVT-TS cells) and purified first-trimester CTBs, STBs, and EVTs [3]. Heatmap representation of relative expression (*Z*-score) of *GCM1* and its differentiation-related target genes *HTRA4*, *PGF*, and *CGB7* as well as *ΔNp63α* and trophoblast stemness genes *ELF5* and *ITGA6* is shown. The ΔNp63α target gene *MTSS1* serves as a positive control. **b,** Meta-analysis of single-cell RNA-seq data of human first-trimester trophoblasts. Single-cell RNA-seq data were extracted from the GSE89497 dataset [4].

The cell no. indicates the single-cell serial number code. Heatmap and dot plot showing the expression levels of the selected genes in individual CTBs, EVTs, and STBs are presented.

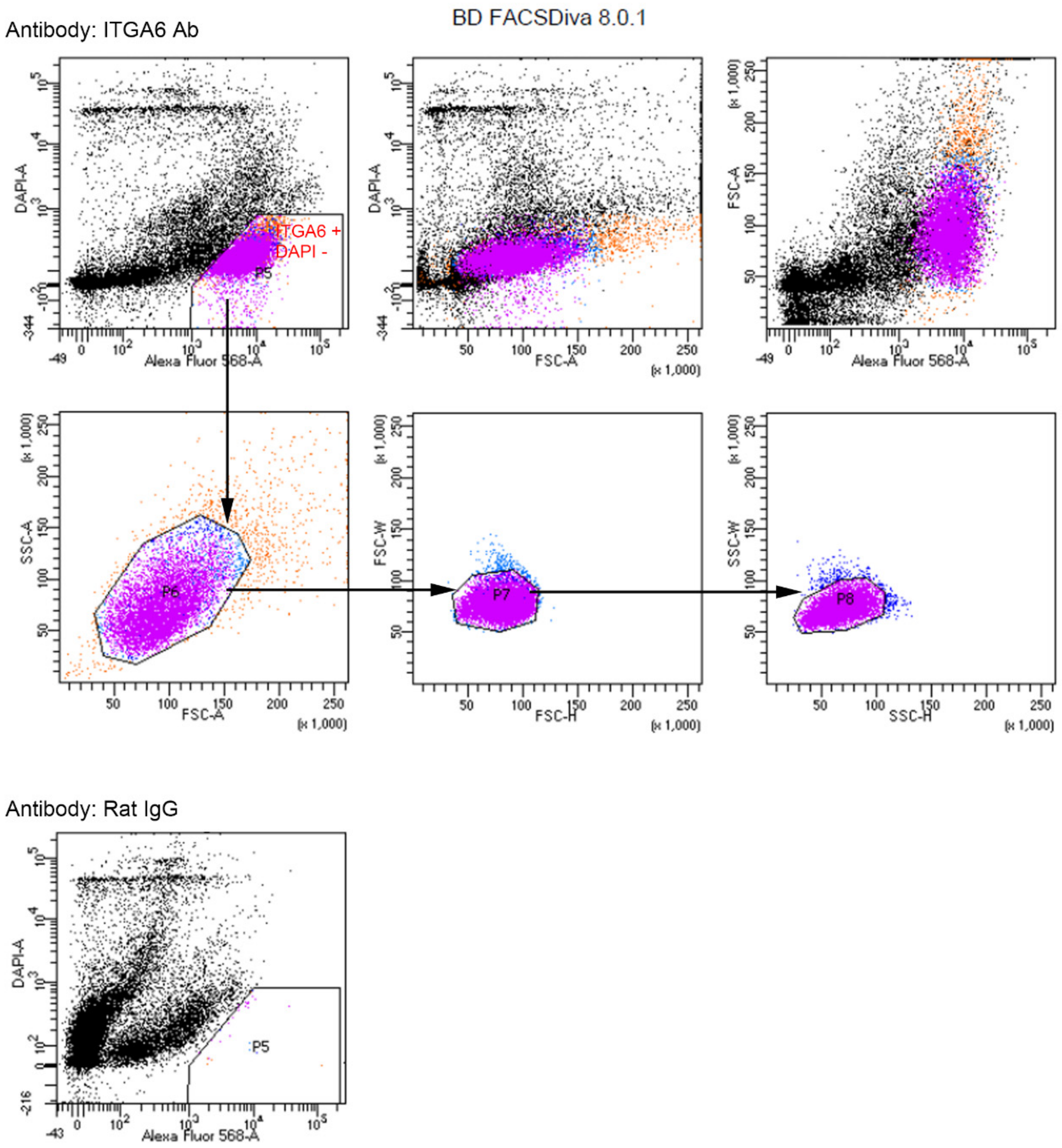

Supplementary Figure 10. Purification of ITGA6-positive cytotrophoblasts from term placentas. Primary trophoblasts were incubated with ITGA6 antibody or normal rat IgG (negative control) and then sequentially gated using Alexa Fluro 588-A/DAPI-A, FSC-A/SSC-A, FSC-H/FSC-W, and SSC-H/SSC-W to remove debris and discard doublets. The preserved singlets were considered as ITGA6-positive and alive cytotrophoblasts.

Supplementary Table 1. List of shRNAs, sgRNAs, and primers

**Gene**	**shRNA target sequence**	
Scramble	CCTAAGGTTAAGTCGCCCTCG	
GCM1	CCTCAGCAGAACTCACTAAAT	
ΔNp63α	AGTTGCACTTATTGACCATTT	
CKMT1A	TGAGGAGACCTATGAGGTATT	
GATA3	CATCCAGACCAGAAACCGAAA	
**Gene**	**sgRNA target sequence**	
GCM1	CAGGAAGGCGTCCAATTGCC	
**Gene (qRT-PCR)**	**Orientation**	**Primer sequence**
ΔNp63α	Forward	AGGAAGAGACAGGAAGGC
ΔNp63α	Reverse	TGTGTGCTGAGGAAGGT
ELF5	Forward	GCTGCGACCAGTACAAGTTG
ELF5	Reverse	CTGCCTCGACGAACTCCTC
EPCAM	Forward	GCCCTCCAGAACAATGATG
EPCAM	Reverse	AGAGCAGGTTATTTCAGTGTC
TEAD4	Forward	GACAGAGTATGCTCGCTAT
TEAD4	Reverse	CTGGCTGACACCTCAAAG
AXIN2	Forward	GTCTCCAAGCAGCTGAAGCC
AXIN2	Reverse	CCTCCATCACCGACTGGATC
GCM1	Forward	AACTCCATCATGAAGTGTGACG
GCM1	Reverse	GATCCACATCTGCTGGAAGG
HTRA4	Forward	TTCTGTGAGCGAGACCC
HTRA4	Reverse	GGAGATTCCATCAGTCACCC
hCGβ	Forward	CTGAGTCTCTGAGGTCACTT
hCGβ	Reverse	TGATAGGATGCTGGGGT
Syncytin-1	Forward	GAAGGCCCTTCATAACCAATGA
Syncytin-1	Reverse	GATATTTGGCTAAGGAGGTGATGTC
PGF	Forward	TCAGAGGTGGAAGTGGTACCCT
PGF	Reverse	GCAGAGGCCGGCATTC
WNT10B	Forward	TTCTGTGAGCGAGACCC
WNT10B	Reverse	CATCACACAGCACATAGC
GATA3	Forward	GACCCACCACCCCATCA
GATA3	Reverse	GGTTCTGTCCGTTCATTTTGT
HLA-G	Forward	CGCACAGACTGACAGAAT
HLA-G	Reverse	AGGTAATCCTTGCCATCGTA
ITGA1	Forward	CCTGTTCTTGATGATTCTCTACC

ITGA1	Reverse	TTGACTGTGAGGCTAACG
FLT4	Forward	ACAACTGGGTGTCCTTTC
FLT4	Reverse	TCTGCTCAAACTCCTCCG
CKMT1	Forward	AAAGATAGCCGCTTCCC
CKMT1	Reverse	GCCGTTCACAATCAATCAAATAGTTTA
UBC	Forward	GCTGGAAGATGGACGCA
UBC	Reverse	ATTCTCAATGGTGTCACTCG
**Gene (ChIP)**	**Orientation**	**Primer sequence**
GATA3	Forward	GAGAAGTCCTGGAGCGG
GATA3	Reverse	TTCTGAAAGTATCAAGACGGC
FGFR2	Forward	AAATGAGCGCGCAAGTTAGA
FGFR2	Reverse	CGAACTGGACCGACTTTTTC
GAPDH	Forward	AAAAGCGGGGAGAAAGTAGG
GAPDH	Reverse	CTAGCCTCCCGGGTTTCTCT

Supplementary references

1. McDade SS, *et al.* Genome-wide analysis of p63 binding sites identifies AP-2 factors as co-regulators of epidermal differentiation. *Nucleic Acids Res* **40**, 7190-7206 (2012).

2. Patturajan M, *et al.* DeltaNp63 induces beta-catenin nuclear accumulation and signaling. *Cancer Cell* **1**, 369-379 (2002).

3. Okae H, *et al.* Derivation of Human Trophoblast Stem Cells. *Cell Stem Cell* **22**, 50-63 e56 (2018).

4. Liu Y, *et al.* Single-cell RNA-seq reveals the diversity of trophoblast subtypes and patterns of differentiation in the human placenta. *Cell Res* **28**, 819-832 (2018).
